# Hypoxia drives hematopoiesis with the enhancement of T lineage through eliciting arterial specification of hematopoietic endothelial progenitors from hESC

**DOI:** 10.1186/s13287-022-02967-0

**Published:** 2022-06-28

**Authors:** Ning Wang, Chuxin Chen, Yang Cheng, Yingjie Fu, Zhiyong Zhong, Yu Yang, Ling Lv, Honglin Chen, Jian Huang, Yuyou Duan

**Affiliations:** 1 School of Biomedical Sciences and Engineering, Guangzhou International Campus, South China University of Technology, Guangzhou, 510006 People’s Republic of China; 2 Laboratory of Stem Cells and Translational Medicine, Institutes for Life Sciences and School of Medicine, Higher Education Mega Center, South China University of Technology, No.382 Waihuan East Road, Suite 406, Guangzhou, 510006 People’s Republic of China; 3 School of Biology and Biological Engineering, South China University of Technology, Guangzhou, 510006 People’s Republic of China; 4 Department of Gynaecology and Obstetrics, Guangzhou First People’s Hospital, Guangzhou, 510180 People’s Republic of China; 5 Research Unit of Liver Transplantation and Transplant Immunology, Chinese Academy of Medical Sciences, Hepatobiliary Center, The First Affiliated Hospital of Nanjing Medical University, 300 Guang Zhou Road, Nanjing, 210029 People’s Republic of China; 6 National Engineering Research Center for Tissue Restoration and Reconstruction, South China University of Technology, Guangzhou, 510006 People’s Republic of China; 7 Guangdong Provincial Key Laboratory of Biomedical Engineering, South China University of Technology, Guangzhou, 510006 People’s Republic of China; 8 Key Laboratory of Biomedical Materials and Engineering of the Ministry of Education, South China University of Technology, Guangzhou, 510006 People’s Republic of China; 9 Coriell Institute for Medical Research, Camden, NJ USA; 10 Cooper Medical School of Rowan University, Camden, NJ USA

**Keywords:** Human embryonic stem cells, Hematopoietic, Hemogenic endothelium, Endothelial-to-hematopoietic transition, Hypoxia

## Abstract

**Background:**

Hematopoietic stem cells are able to self-renew and differentiate into all blood cell lineages. Hematopoietic stem cell transplantation is a mainstay of life-saving therapy for hematopoietic malignancies and hypoproliferative disorders. In vitro hematopoietic differentiation of human pluripotent stem cells (hPSCs) is a promising approach for modeling hematopoietic development and cell replacement therapies. Although using hPSCs to derive hematopoietic progenitor cells has achieved some successes in the past, differentiation from hPSCs to produce all hematopoietic cells which can provide robust long-term multilineage engraftment is still very difficult. Here, we reported a novel culture system for hematopoietic differentiation from human embryonic stem cells (hESCs) with optimal cytokines combinations under hypoxia condition.

**Methods:**

In vitro production of T lineage hematopoietic stem/progenitor cells from hESCs by using hypoxia differentiation system, the effects and the potential mechanism of hypoxia promoting T lineage hematopoiesis were investigated by RT-qPCR validation, cell cycle assay and flow cytometry analysis.

**Results:**

Using our differentiation system, almost 80% CD45^+^ cells generated from hESCs were hematopoietic cells and particularly could be further induced into CD3^+^TCRαβ^+^ T cells in vitro. We detected more CD34^+^CD144^+^ hematopoietic endothelial progenitors (HEPs) induced from hESCs than those in normoxia conditions, and the early HEPs-related gene DLL4 was upregulated by enhancing the hypoxia signaling via potential HIF-1α/NOTCH1/DLL4 axis to enhance arterial feature, thus drove T lineage during the hematopoiesis. Strikingly, hematopoietic cells generated in our system exhibited the potential for all multilineage reconstruction including lymphoid, myeloid and erythroid lineages in vivo by transplantation assay.

**Conclusion:**

Our results demonstrated that hypoxia plays an important role in T lineage hematopoiesis by promoting the expression of arterial endothelial gene DLL4 and upregulation of NOTCH1 through the activation of the HIF-1α signaling pathway. These results provide a significant approach for in vitro and in vivo production of fully functional hematopoietic stem/progenitor cells from hESCs.

**Supplementary Information:**

The online version contains supplementary material available at 10.1186/s13287-022-02967-0.

## Introduction

Hematopoietic stem cells (HSCs) have the capability to self-renew and differentiate into all blood cell lineages. Several decades of successful HSC transplantations (HSCT) have demonstrated the therapeutic importance of HSCs [1–3]. However, a major limitation is that the supply of HSCs provided by donors is not enough to meet large clinical demand and it seriously restricts the clinical application of HSCT [4]. Hematopoietic cells derived from human pluripotent stem cells (hPSCs) not only provided a novel alternative strategy for the source of transplantation, but also present a new model for the study of hematopoietic development [5, 6]. However, to date, differentiating hPSCs to functional HSC with multilineage reconstruction capability, especially T lineages, has been challenging. Even though many studies have focused more on forcing expression of hematopoietic transcription factors or supplying exogenous cytokines to induce hematopoietic fate enhancement to drive T lineage regeneration [7, 8], low safety and accuracy of gene editing have limited their clinical applications. Exogenous cytokines did drive the fate of the hematopoietic lineage to some extent [9, 10]; however, those hematopoietic cells could not effectively reconstruct the hematopoietic system after transplantation. Therefore, it is still difficult to derive therapeutic HSCs from hPSCs ex vivo.

One key determinant for the fate choice of the hematopoietic lineages is the specification of hematopoietic endothelial progenitors (HEPs), precursors of blood cells with transient endothelial properties derived from the lateral group of embryonic mesoderm forming the vascular system [11–14]. Although current hematopoietic differentiation system provides a valuable insight for better dissecting and mimicking the in vivo pathophysiology [9, 10], the investigation on the specification of hematopoietic has been hampered by a lack of consensus on the timing of HEPs and the hematopoietic potential analysis. Several studies have found that the previous differentiation system could only achieve low hematopoietic cell production and T lineage reconstruction defects, which significantly decreased in the efficacy of transplantation [5, 7, 8]. Based on this, we aimed to optimize the in vitro differentiation system by regulating the role of exogenous cytokines to improve the yield of hematopoietic cells derived from hPSCs. Previous study has also provided compelling evidence that despite the gradual downregulation of oxygen metabolism, the endothelial-to-hematopoietic transition (EHT) in vitro is still in an active state of aerobic metabolism compared with the in vivo data of the fetal liver and bone marrow, which identified as an important molecular feature during the EHT stage [15].

In addition, normal cell culture conditions around the world are performed to use with high oxygen concentrations far beyond physiological conditions [16–18]. Even brief exposure could have a significant negative effect on HSCs, resulting in a decrease in the number and function of HSCs and ultimately a decrease in the efficacy of transplantation [19, 20]. HIF-1α, a key transcription factor of cells in the response to hypoxia [21, 22], not only is an important molecular feature during the EHT stage [15], but also interferes with mitochondrial aerobic metabolism and decreases ROS production [23], thereby repressing HSC apoptosis [15, 19]. Studies of HSCs with multi-lineages observed in mouse embryos have shown that HIF-1α effectively binds to a common sequence at promoters or enhancers of a series of downstream target genes whose function is usually associated with HSC maintenance in bone marrow niches, such as EPO, VEGFA, and SDF-1/CXCL12 [24]. Therefore, hypoxic conditions are critical for hematopoiesis and introduced into our differentiation system.

Considering the important role of hypoxia in the maintenance of HSCs function, we attempted to evaluate the regulatory role of hypoxia in optimized hematopoietic differentiation system, with a view to achieving both yield and function enhancement effects. Using the optimized hematopoietic differentiation system in vitro as a model for human embryonic hematopoiesis, we can not only obtain 80% CD45^+^ hematopoietic cells, but also further improve the induction efficiency of CD3^+^TCRαβ^+^ T cells. By comparing in vivo and in vitro data, we confirmed that hypoxia was sufficient to promote hematopoiesis during the hematopoietic differentiation of hPSCs and further provided the evidence that the EHT was dependent on activation of hypoxia signaling via enhancing arterial feature, thus drove T lineage during the hematopoiesis.

## Materials and methods

### Human ESC cultivation

The H9 hESCs (WA09) were obtained from the WiCell Research Institute (Madison, WI, USA) with the Material Transfer Agreement (No.19-W0512) and cultured on mitomycin-treated mouse embryonic fibroblasts (MEFs) from CF-1 mice in hESC culture medium (DMEM/F12 medium, 20% knock out serum replacement, 1 mM L-glutamine, 1% NEAA, 1% penicillin/streptomycin (all from Invitrogen), 0.1 mM β-Mercaptoethanol (Sigma-Aldrich) and 10 ng/mL bFGF (Peprotech). The hESCs were subcultured every 5–6 days with a treatment of collagenase IV for passaging.

### Human ESC differentiation

Single-cell suspensions of H9 hESCs were obtained by treating hESCs by Gentle Cell Dissociation Reagent (GCDR) (STEMCELL), and then mesodermal EBs were generated on the ultra-low attachment 6-well plates with APEL medium (STEMCELL Technologies) supplemented with 20 ng/mL BMP4, 10 ng/mL Activin A, 25 ng/mL VEGF, 10 ng/mL SCF, 10 ng/mL bFGF (Peprotech), 3 μM CHIR99021, 4 μM SB431542 and with 10 μM Rock inhibitor (Y-27632; STEMCELL Technologies). At day 4, the culture medium was removed and fresh APEL medium containing 20 ng/mL BMP4, 50 ng/mL VEGF, and 10 ng/mL bFGF, 10 ng/mL SCF and 15 ng/mL IGF2 was replenished until day 7. The EBs at day 7 were collected and plated on Matrigel-coated wells to differentiate into HEPs in the presence of APEL differentiation medium I supplemented with 50 ng/mL IL-3, 100 ng/mL SCF, 25 ng/mL Flt3L, 50 ng/mL VEGF, 25 ng/mL IL-6, 25 ng/mL TPO, 3U/mL EPO, 10 ng/mL bFGF and 20 ng/mL IGF2. From day 11, the last step of the hematopoietic differentiation was carried out in APEL differentiation medium II supplemented with 50 ng/mL VEGF, 100 ng/mL SCF, 25 ng/mL IL-6, 25 ng/mL TPO, 25 ng/mL Flt3L, 10 ng/mL bFGF and 20 ng/mL IGF2 for 4 more days. Cultures were maintained at 37 °C under the normoxia or hypoxia (5% O_2_) conditions as indicated.

### Analysis of mesodermal progenitor cells

hESCs and EBs at days 2 and 4 were dissociated into single-cell suspension by TrypLE (Thermo Fischer Scientific), and then cells were incubated with antibodies against human EpCAM and CD56 antibodies. Finally, DAPI (5 mg/mL) was added to gate out dead cells and flow cytometry was performed on a BD FACSCanto II (BD Biosciences) and the data were analyzed using FlowJo Software (Version 10).

### Cell surface analysis

In order to perform flow cytometry analysis, hESCs and EBs at days 4 and 7, adherent and suspension cells at days 11 and 15 after the differentiation were dissociated into single-cell suspension by TrypLE (Thermo Fischer Scientific), and then cells were washed with PBS containing 0.1% FBS and stained with antibodies against human CD34, CD31, KDR, CD45 and CD144 antibodies as per manufacturer’s protocol. Dead cells were excluded from the analysis by staining with 7-AAD (Miltenyi Biotec). Finally, flow cytometry was performed on BD FACSCanto II (BD Biosciences) and the data were analyzed using FlowJo Software (Version 10). All antibodies used are listed in Additional file 1: Table S1.

### Real-time quantitative polymerase chain reaction (RT-qPCR)

Total RNA was extracted using RNAiso Plus (Takara, 9109) according to the manufacturer’s manuals. RNA concentrations were determined using a UV spectrophotometer (NanoDrop One) at an absorbance ratio of 260 to 280 nm (A260/280). Reverse transcription was performed using a PCR machine (Veriti 96). All qPCR experiments were performed on a real-time PCR machine (ABI, ABI7500) with the QuantiTect SYBR Green PCR Kit (Takara) and specific primers of the genes of interest. Gene expression was quantified by the comparative cycle threshold (Ct) method. The relative amounts of target gene expression were determined by subtracting the Ct values of these genes from the Ct value of the housekeeping gene GAPDH (ΔCt). The data were presented as 2^−(ΔΔCT)^. The primer sequences used in this study are listed in Additional file 1: Table S2.

### Cell colony-forming assay

hESC-derived CD45^+^ cells at day 15 after differentiation and CB CD34^+^ cells were collected and seeded into 1 mL MethoCult H4435 (STEMCELL Technologies) for the colony forming at a frequency of 5000 cells per dish and supplemented with 10 ng/mL Flt3L, 10 ng/mL IL-6 and 50 ng/mL TPO (PeproTech). Hematopoietic colonies containing more than 50 cells were scored 14 days after the colony forming, and then the colony-forming cells (CFC) were collected for Wright–Giemsa staining.

### Analysis of CFC cells

CFCs were collected and washed with PBS for three times, and then cells were incubated with the following antibodies (5μL/1 × 10^6^ cells) against human CD45, CD235a, CD16 and CD11b. DAPI (5 mg/mL) was added to gate out dead cells. Flow cytometry was performed on a BD FACSCanto II (BD Biosciences), and the data were analyzed using FlowJo Software (Version 10). The expression of CFC-related genes was evaluated by RT-qPCR analysis with the QuantiTect SYBR Green PCR Kit (Takara) and specific primers of the genes of interest. Gene expression was quantified and presented as 2^−(ΔΔCT)^. The primer sequences used in this study are listed in Additional file 1: Table S2.

### Immunofluorescent staining

The EBs, adherent and suspension cells were collected and fixed in 4% PFA at room temperature for 15 min. Subsequently, the cellular membranes were broken with blocking buffer that contained 0.2% Triton X-100 (Sigma-Aldrich, T8787) and nonspecific proteins were blocked with 3% normal goat serum (Jackson Immuno Research, 017–000-121) in PBS at room temperature for 45 min. Cells were incubated with primary antibodies at 4 °C overnight and next day incubated with secondary antibodies (CST, 8890S, 8889S, 4408S and 4412S). Finally, the cell nuclei were counterstained with DAPI for 10 min and imaged using CLSM (Ni-E-A1, Japan).

### ***Isolation of human CD34***^+^***cells from umbilical cord blood***

Fresh umbilical cord blood (CB) of human healthy donors was collected from Guangzhou First People's Hospital; this study was supervised and approved by the ethics committees at Guangzhou First People's Hospital and South China University of Technology (No.: K-2021-008-01). Human peripheral blood mononuclear cells (PBMC) were separated from CB by density gradient Ficoll-Histopaque isolation. Human CD34^+^ cells were isolated from PBMC using the EasySep human CD34 positive selection kit (STEMCELL Technologies, Cat# 18,056). DAPI^−^CD34^+^ cells were then further sorted for transplantation.

### *Induction of T cells *in vitro

For T cell differentiation [15], 5 × 10^5^ hESC-derived CD45^+^ cells were centrifuged with 1 × 10^6^ OP9 cells (derived from neonatal OP/OP mouse cranium for supporting the production of T cell) to form small 3D aggregates, which were then plated onto a 0.4-mm Millicell transwell insert (EMD Millipore) placed in a six-well plate containing 1 mL of T cell differentiation medium consisting of RPMI 1640 (Gibco), 4% B27 supplement (Thermo Fisher Scientific), 30 mM l-ascorbic acid 2-phosphate sesquimagnesium salt hydrate (Sigma-Aldrich), 1% penicillin/streptomycin (Thermo Fisher Scientific), 20 ng/mL stem cell factor (SCF), 5 ng/mL FLT3L, 5 ng/mL IL-7 and 10 ng/mL DLL4 (all from PeproTech). Differentiation medium was changed every 2 days and cultured for 8 weeks. Small 3D aggregates consisting of CB CD34^+^ cells and OP9 cells were cultured as above described. The CD3^+^ TCRαβ^+^ cells were then analyzed by flow cytometry on the days indicated.

### Mouse transplantation and analysis of engraftment

NOD-*Prkdc*^*em26Cd52*^*Il2rg*^*em26Cd22*^*kit*^*em1Cin(V831M)*^/Gpt immunodeficient female mice (NCG-X; 6–8 weeks old) purchased from GemPharmatech Company (Nanjing, China) were housed under sterile conditions. NCG-X mice were intravenously inoculated with 3 × 10^5^ CB CD34^+^ cells or hESC-derived CD45^+^ cells were performed as previously described [7]. After the indicated time, the hematopoietic reconstitution was assessed by FACS analysis of peripheral blood (PB) and bone marrow (BM) tissues. The cells harvested from PB and BM were stained with a cocktail of antibodies against CD45, CD235a, CD16, CD11b; CD45, CD56, CD19, CD3. Dead cells labeled with DAPI (Sigma) were excluded. After incubation for 30 min at 4 °C in the dark, cells were washed twice with PBS. The analysis of the cell surface phenotype was performed by BD FACSCanto II (BD Biosciences), and the data were analyzed using FlowJo Software (Version 10). All antibodies used are listed in Additional file 1: Table S1.

### Generation of engineered hESCs with the EGFP and mCherry reporters

A 1.2 kb subfragment of the HOXA9 promoter (cloned from genomic DNA of LO2 cells) was inserted into a lentiviral vector harboring a mCherry reporter gene, in which the EGFP reporter gene was driven by universal EF1α promoter. hESCs were transduced with the lentiviral virus designated as EF1α-EGFP-HOXA9-mCherry construct with a multiplicity of infection (MOI) of 10 as single cells in feeder-free culture. EGFP-positive cells were then sorted and cultured on mitomycin-treated MEFs in hESC culture medium for several passages. Subsequently, the hematopoietic differentiation was recorded by living cell tracer system (JuLI Stage, Korea), and photographs were taken at the indicated time points.

### HEPs sorting

The EBs at day 7 were dissociated into single cell using TrpLE (Thermo), filtered through a 70-μm filter and stained with anti-human CD34 antibody conjugated with PE, anti-human CD31 antibody conjugated with AF700 and anti-human CD45 antibody conjugated with APC/Cy7 for 30 min at 4 °C; finally, CD34^+^CD31^+^CD45^−^HEPs were isolated by FACS.

### Immunofluorescent staining of HEPs

2 × 10^4^ sorted hESC-derived CD34^+^CD31^+^CD45^−^ cells and human vascular endothelial cells (HUVECs) were cultured in endothelial cell growth medium-2 (Lonza). Cells were collected and incubated with anti-VE-Cadherin antibody (1:1000, Stem Cell, 2500S) at 4 °C overnight and then incubated with secondary antibodies (CST, 4412S). Finally, cells were counterstained with DAPI (Sigma) for 10 min and imaged using CLSM (Ni-E-A1, Japan).

### Vascular-like network formation assay

2 × 10^4^ sorted hESC-derived CD34^+^CD31^+^CD45^−^ cells and HUVECs were immediately cultured in semisolid Matrigel for tube formation assay. Inverted-phase contrast microscope (Nikon, Japan) was taken to observe the tube formation after 6 h.

### Analysis for cell cycle by flow cytometry

CD45^+^ cells at day 15 differentiated under the normoxia and hypoxia were collected and washed twice with PBS, and then 75% precooled ethanol was added and fixed overnight for 4 °C. RNAase was dripped and incubated at 37 °C for 30 min. After washed with PBS, cells were labeled with propidium iodide (PI) for 20 min. Finally, PBS was supplemented and cell cycle was detected by flow cytometry.

### Immunofluorescence staining for ROS1

CD45^+^ cells at day 15 differentiated under the normoxia and hypoxia were collected for the immunofluorescent staining. Cells were incubated with anti-ROS1 antibody (1:400, Bioss, bs-2504R) at 4 °C overnight and then incubated with secondary antibodies (CST, 8889S). Finally, cells were counterstained with DAPI (Sigma) for 5 min and imaged using CLSM (Ni-E-A1, Japan).

### Statistical analysis

The data were subjected to statistical analysis by the Student’s t test. Results with a value of *p* < 0.05 were considered statistically significant.

## Results

### Embryonic hematopoietic mesoderm development was recapitulated during the hematopoietic differentiation from hESCs

**Fig. 1:**
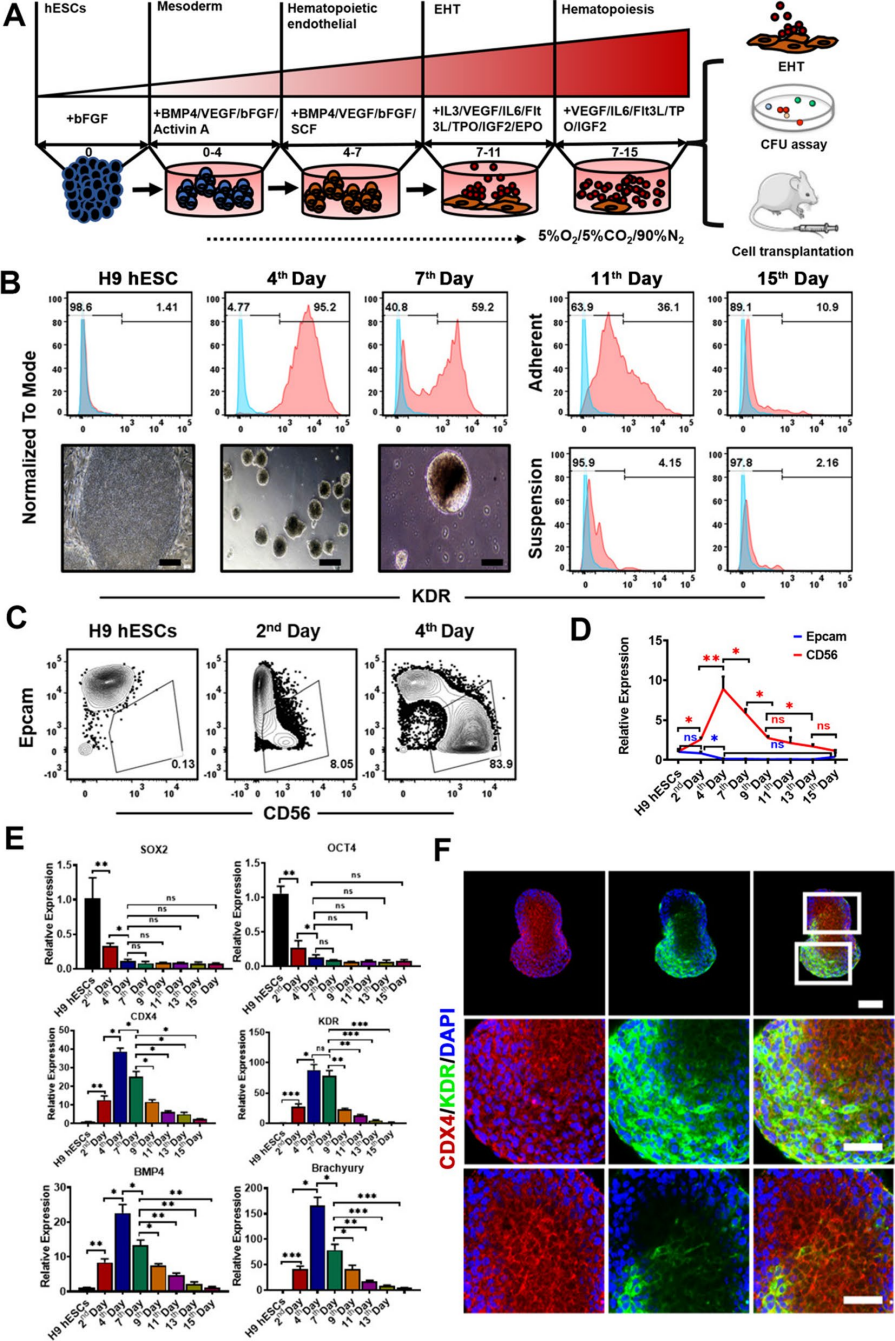
Identification of mesodermal specification during the hematopoietic differentiation from hESCs. **A** Schematic representation for the generation of definitive HPCs from hESCs. **B** The expression of mesoderm surface marker (KDR) and corresponding isotype controls (shown in blue) during the in vitro hematopoietic development from hESCs. The lower left panel showed representative images of EBs at different time points. (Original magnification, 10×, Scale bar, 100 μm). **C** Generation of EpCAM^−^CD56^+^ mesoderm-enriched cells shown by flow cytometry at days 0, 2 and 4 employing hematopoietic differentiation protocol. **D** Relative expression levels of EpCAM and CD56 genes were determined and quantified by RT-qPCR in the bar graphs. **E** Expression analysis of pluripotent genes (SOX2 and OCT4) and mesodermal genes (CDX4, KDR, BMP4 and Brachyury) for different cell populations at different time points during the hematopoietic specification from hESCs by RT-qPCR. Data represent the mean ± SEM. *p < 0.05, **p < 0.01, ***p < 0.001 and ns (no significance). **F** EBs were fixed with PFA and subjected to immunofluorescent staining for KDR and CDX4 at day 4 of differentiation from hESCs. (Original magnification, 20×, scale bar 40 μm, upper panel; 40×, scale bar 50 μm, lower panels.)

To explore the regulatory process of the hematopoietic differentiation from hESCs, we developed a four-stage hematopoietic differentiation system under hypoxia condition, which promoted the generation of hematopoietic lineages from hESCs (Fig. 1A). At present, hematopoietic lineage cells differentiated from hPSCs could generate various types of blood cells in vivo and in vitro, such as granulocytes or erythrocytes [5, 6], but the insufficient production of lymphoid lineage indicated that the function of those hematopoietic progenitor cells (HPCs) still had certain defects. Based on the importance of hypoxia environment in embryonic development and adult hematopoietic site in zebrafish and mammals, we sought to use hypoxia condition (5% O_2_) to study in vitro embryonic hematopoiesis. Since both waves of hematopoiesis were derived from the lamellar cells of lateral mesoderm, in order to verify the induction effect on hESC-derived mesodermal differentiation, we first quantified the proportion of mesodermal KDR^+^ cells by flow cytometry (Fig. 1B). We found that a larger proportion of KDR^+^ cells were measured at day 4 upon the differentiation from hESCs, reflecting the generation of mesodermal lineages. Moreover, the earliest defined mesodermal progenitors derived from hESCs were identified by the expression of neuronal cell adhesion molecule (NCAM/CD56) and no expression of epithelial cell adhesion molecule (EpCAM/CD326). Flow cytometry analysis showed that almost 83% EpCAM^−^CD56^+^ cells were recognized at day 4 (Fig. 1C). At the same time, this result was also independently verified by RT-qPCR (Fig. 1D), indicating that CD56 was at highest expressed at day 4 and downregulated overtime. The analysis of gene expression pattern also showed that the developing embryoid bodies (EBs) mimicked the process of in vivo mesodermal development by expression of the primitive streak/mesendoderm genes including Brachyury (T), KDR, BMP4 and CDX4, which were observed and peaked on the 4th day and subsequently downregulated progressively along with the hematopoietic specification (Fig. 1E). On the other hand, the expression of pluripotent genes, SOX2 and OCT4, was dramatically decreased during the mesodermal development after the differentiation (Fig. 1E). Coincident with these aforementioned results of flow analysis and RT-qPCR, immunofluorescence staining also showed the high expression of mesendodermal markers, like CDX4, KDR, Brachyury and SOX17 in EBs of the 4th day (Fig. 1F and Additional file 1: Fig. S1). Collectively, these results showed a progressive loss of pluripotency and the acquisition of mesoderm during the hematopoietic mesoderm development from hESCs.

### Characterization of hESC-derived HEPs during the hematopoietic specification from hESCs

**Fig. 2:**
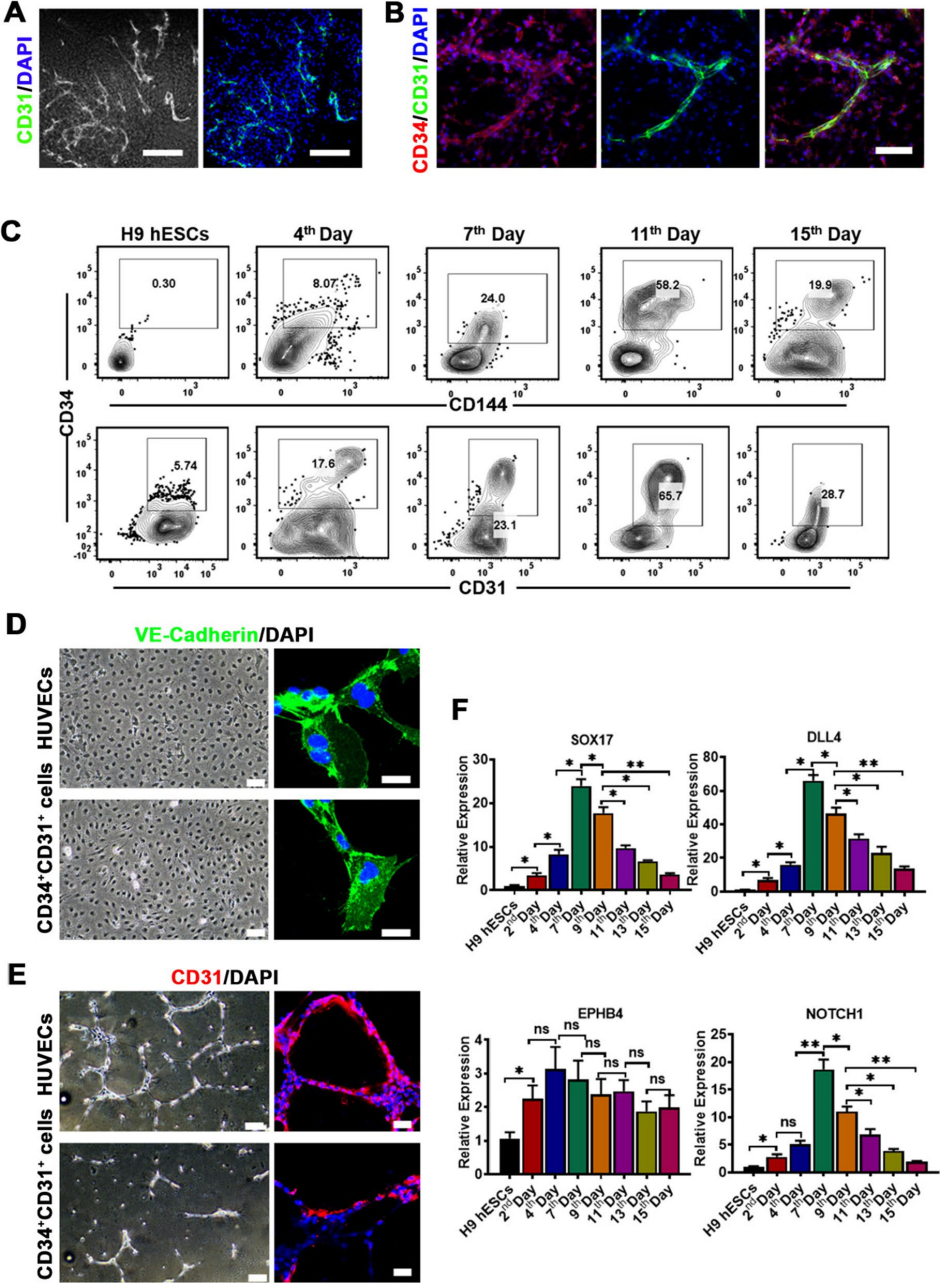
Characterization of hESC-derived HEPs by analysis of gene expression pattern and structural features during the development of hematopoiesis after the differentiation. **A** Fluorescence microscopy of developing the EBs at day 7 after the differentiation (bright field (left panel) and the merge of immunofluorescence staining (right panel), the colors of green and blue represent the staining for CD31 and nuclei, scale bar, 40 μm). **B** CLSM images of the immunostaining for CD34 and CD31 in the EBs at day 7 after the differentiation. The colors of green, red and blue represent the staining for CD31, CD34 and nuclei. (Original magnification, 20×, scale bar 40 μm). **C** Representative flow cytometry results showed the population of CD34^+^CD144^+^ and CD34^+^CD31^+^ HEPs at different days during the hematopoietic specification after the hematopoietic differentiation from hESCs. **D** Morphology of HUVECs and HEPs at day 6 post-sorting and cultured in EGM2 medium (10× magnification, left panel), and CLSM images of the immunostaining for VE-Cadherin. The colors of green and blue represent the staining for VE-Cadherin and nuclei. (Right panel, original magnification, 40×, scale bar 50 μm). **E** Tube formation by HUVECs and HEPs on Matrigel-coated plate in EGM2 medium. (20,000 cells/well in 12-well culture plates, photographs taken 12 h after the culture; 10× magnification, left panel). CLSM images of the immunostaining for CD31. (Right panel, 40×, scale bar 50 μm). **F** Gene expression analysis for arterial genes (SOX17, DLL4 and NOTCH1) and venous gene (EPHB4) during the development of the hematopoiesis after the hematopoietic differentiation from hESCs. Data represent the mean ± SEM. *p < 0.05, **p < 0.01, and ns (no significance)

In order to further explore embryonic hematopoiesis in vitro, it is extremely essential to evaluate the expression of stage-specific markers that are identified to regulate EHT in the early embryonic hematopoiesis. Vascular branch-like endothelial cells were formed within the EBs of the 7th day, which expressed CD31 determined by immunofluorescence staining (Fig. 2A). To further confirm its hematopoietic endothelial properties, we performed double immunofluorescent staining with the EBs of the 7th day using endothelial cell-specific markers, CD31 and CD34 (Fig. 2B). Results revealed strongly elevated co-expression for CD31 and CD34 in the rim of the EBs, which indicated HEPs. Moreover, surface antigens such as CD144 and CD34, CD31 and CD34, were co-expressed on HEPs, which were first detected from the 4th day (17.6 ± 0.35% for CD31^+^CD34^+^cells and 8.07 ± 0.72% for CD144^+^CD34^+^cells, mean ± SEM, *n* = 4) and dramatically increased at the 7th day (23.1 ± 0.29% for CD31^+^CD34^+^cells and 24.0 ± 0.57% for CD144^+^CD34^+^cells, mean ± SEM, *n* = 4) and peaked in anchorage-dependent cells until the 11th day (65.7 ± 3.81% for CD31^+^CD34^+^cells and 58.2 ± 3.16% for CD144^+^CD34^+^cells, mean ± SEM, *n* = 4) (Fig. 2C). Henceforth, a significant reduction in HEPs population was detected at day 15 (Fig. 2C). When purified from the EBs of the 7th day by FACS, CD31^+^CD34^+^CD45^−^HEPs could be cultured in EGM2 medium which is used for the culture of the endothelial cells for more than two passages, and showed similar endothelial morphology to HUVEC (Fig. 2D). As shown in Fig. 2D, E, HEPs were able to exhibit tube-forming activity on Matrigel and highly expressed CD31 and VE-Cadherin as determined by immunofluorescent staining. Genes of the arterial endothelium, including SOX17, DLL4 and NOTCH1 (Fig. 2F), started to be expressed, whereas the venous endothelial gene, like EPHB4, relatively lowly expressed (Fig. 2F). It is well known that the definitive HSCs (but not the primitive ones) are derived from endothelial cells of the dorsal aorta rather than from the vein endothelium.

### Hematopoietic differentiation under hypoxia condition demonstrated embryonic EHT development in vitro

**Fig. 3:**
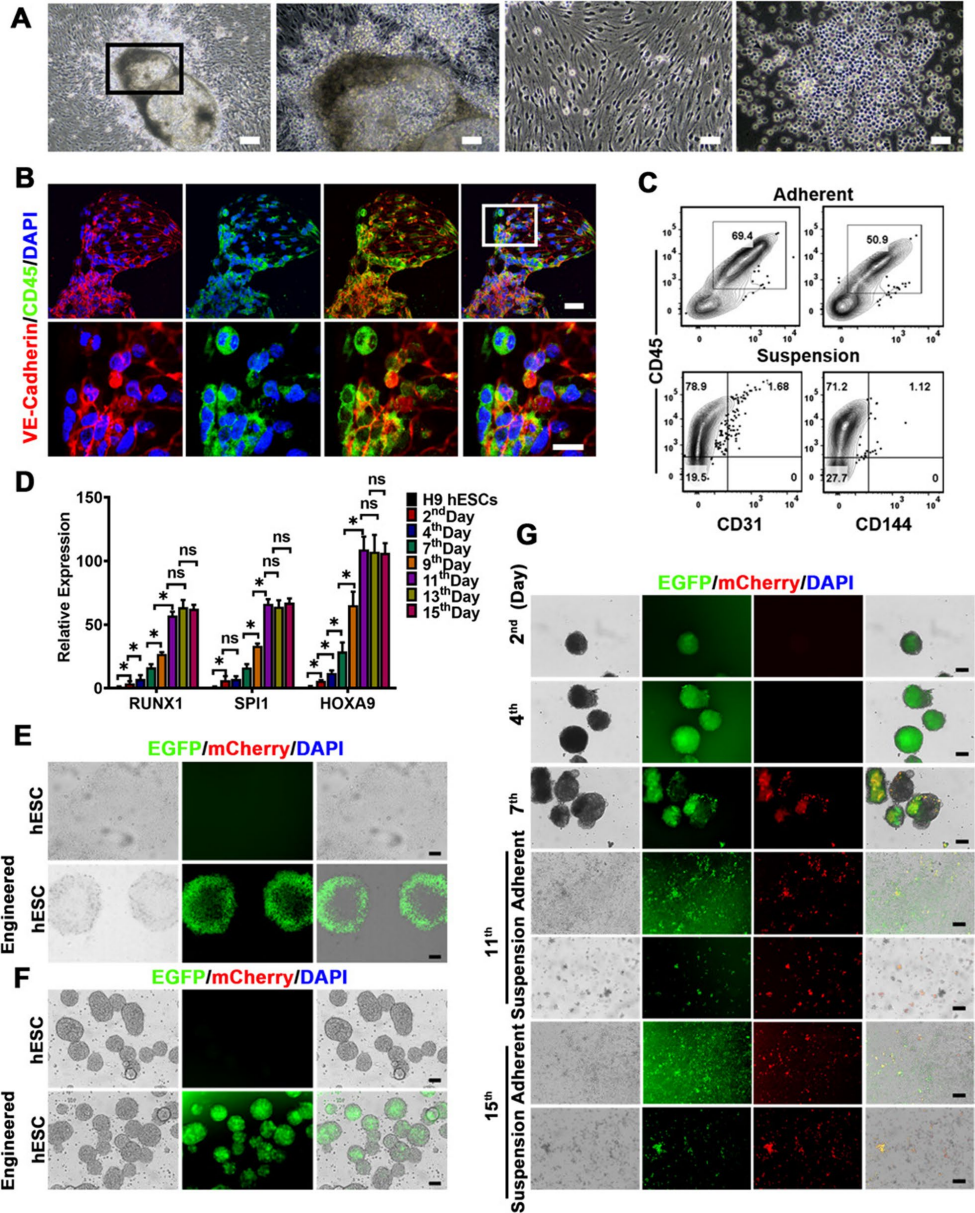
Characterization of the occurrence of the EHT during the hematopoietic specification. **A** Morphological features of adherent cells and suspension cells climbed out from aforementioned adherent cells at the 11th day after the hematopoietic differentiation from hESCs (10× magnification, scale bars 100 μm, far left one photo; 20× magnification, scale bars 50 μm, right three photographs). **B** CLSM images of the immunostaining for VE-Cadherin and CD45 in the differentiated cells at day 11 undergoing the EHT. The colors of green, red and blue represent the staining for CD45, VE-Cadherin and nuclei, respectively. (Original magnification, 20×, scale bar 40 μm, upper panel; 40×, scale bar 50 μm, lower panel.). **C** Flow analysis for HEPs (CD31^+^CD45^+^ cells or CD144^+^CD45^+^ cells) at the 11th day during the hematopoiesis. **D** The dynamic gene expression of the indicated HSC-specific transcription factors by RT-qPCR during the hematopoiesis. **E** The transduced efficiency of lentivirus containing EGFP and mCherry reporters in hESCs was evaluated by fluorescent microscopy. (Original magnification, 10×, scale bar 100 μm). **F** The aggregate-forming ability of engineered hESCs seeded on the low adhesion plate was not affected by the transduction with the lentivirus. (Original magnification, 4×, scale bar 10 μm) **G** The generation of EGFP and mCherry double-positive hematopoietic cells during the development of the hematopoiesis was visualized by living cell fluorescence trace system. (Original magnification, 4×, scale bar 100 μm). Data represent the mean ± SEM. *p < 0.05 and ns (no significance)

Given that EHT is a critical stage for hematopoietic development, we then paid our attention to the EHT process during the hematopoiesis by using the hematopoietic differentiation system in vitro. The EBs of the 7th day seeded on Matrigel resulted in the formation of characteristic of cobblestone areas and round-shaped suspension cells approximately 4 days post-culture, showing the typical changes of cellular morphology and the switch of cultural feature from the adherent cells to suspension state, which is essential for undergoing the EHT (Fig. 3A). Immunofluorescent staining further confirmed the occurrence of the EHT during these changes after the culture of the EBs on Matrigel as we detected CD45^+^VE-Cadherin^+^ HEPs (Fig. 3B). These outcomes were consistent with those previously reported in the literature [11–14]; the first HSC emerging in the AGM bears a distinctive dual hematoendothelial immunophenotype co-expressing both VE-Cadherin and RUNX1 or CD45 markers. Analysis of the phenotype of these cells revealed that the endothelium within these clusters gradually acquired hematopoietic morphology and phenotype and consequently underwent EHT. The adherent cells and suspended cells at the 11th day after the differentiation were collected and evaluated for markers associated with hematopoietic endothelium/hematopoietic progenitor lineage, the results of the flow cytometry analysis showed that the population of HEPs was 69.4% for CD45^+^CD31^+^cells and 50.9% for CD45^+^CD144^+^cells in the adherent cells, and almost 80% of the population was hematopoietic cells with CD45^+^cells in suspended cells, revealing that HEPs lost their endothelial identity and acquired hematopoietic characteristics during the changes of the morphology and cultural feature, which strongly supported the occurrence of the EHT (Fig. 3C). Moreover, accompanied with hematopoietic transition, HSC-specific transcription factors were highly expressed from the 11th day after the induction of hematopoiesis from hESCs (Fig. 3D). It has been reported that HOX family genes, especially the most highly expressed HOXA9, have important roles in hematopoiesis, and specifically regulate the EHT progression [25]. To investigate EHT potential in our system of in vitro differentiation, we developed a lineage tracing model in which HOXA9-expressing cells were labeled with a fluorescent reporter (EF1α-EGFP-HOXA9-mCherry) (Additional file 1: Fig. S2A). The hESCs were transduced with the lentivirus containing universal EF1α promoter to drive the EGFP for evaluating transfection efficiency, wherein mCherry fluorescence was derived by hematopoietic specific HOXA9 promoter to trace hematopoietic cells. The pluripotency and morphology of engineered hESCs were not disturbed, as determined by the culture of EGFP^+^ hESCs under 2D and 3D conditions and the flow cytometry analysis for pluripotent genes OCT4 and SSEA4 (Fig. 3E, F, Additional file 1: Fig. S2B, C). Small amounts of cells co-expressed EGFP (green) and mCherry (red) were visualized from the 7th day after the hematopoietic differentiation (Fig. 3G). As hematopoietic differentiation proceeded, more EGFP/mCherry double-positive round cells suspended in the medium were observed from the 11th day, clearly showing that the hematopoietic-like cells with double-positive cells gradually climbed out to flow in the culture medium from network-like structure of the adherent cells (Fig. 3G). Together, these timely visualized results further affirmed their EHT identity in vitro.

### Hematopoietic differentiation in vitro recapitulated embryonically hematopoietic development

**Fig. 4:**
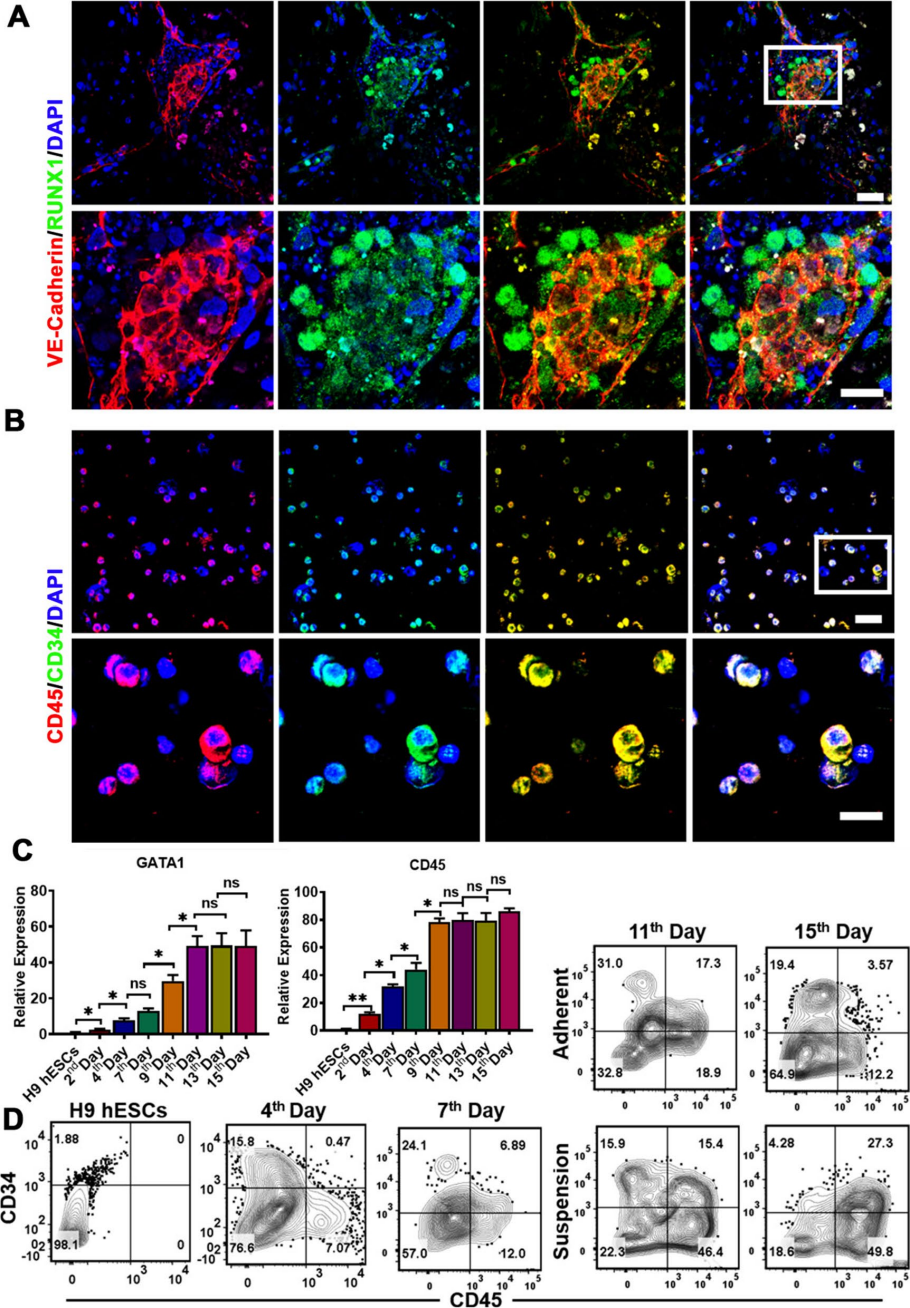
Characterization of hematopoietic specification after the hematopoietic differentiation from hESCs. **A** CLSM images of the immunostainings for VE-Cadherin and RUNX1 in adherent cells at day 15 during the hematopoietic specification after the differentiation. The colors of green, red and blue represent the staining for RUNX1, VE-Cadherin and nuclei. (Original magnification, 20×, scale bar 40 μm, upper panel; 40×, scale bar 50 μm, lower panel). **B** CLSM images of the immunostainings for CD45 and CD34 in suspension cells at day 15 during the hematopoietic differentiation. The colors of green, red and blue represent the staining for CD34, CD45 and nuclei. (Original magnification, 20×, scale bar 40 μm, upper panel; 40×, scale bar 50 μm, lower panel). **C** Analysis of hematopoietic gene expressions at defined stages during the differentiation by RT-qPCR (*n* = 3, mean ± SEM). **D** Flow analysis for HPCs (CD34^+^CD45^+^ cells) during the hematopoietic development after the differentiation. Data represent the mean ± SEM. *p  < 0.05, **p < 0.01, and ns (no significance)

In order to further delineate the hematopoietic specifications, we next investigated the hematopoietic cells at day 15 after the differentiation as more endothelial cells turned into hematopoietic cells at this stage. Immunofluorescent staining of HEPs clusters revealed that the gradual acquisition of the morphology and phenotype RUNX1^+^ hematopoietic cells from VE-Cadherin^+^ aortic endothelial cells at day 15 (Fig. 4A) eventually acquired a round shape characteristic of blood cells (Fig. 4B). Importantly, we observed the high expression of GATA1 and CD45 in suspension cells from the 9th day by RT-qPCR (Fig. 4C), demonstrating that hematopoietic cells subsequently were generated. Moreover, the emergence of the hematopoietic cells characterized by expression of CD34 and CD45, at the first time, was observed at day 11, as determined by immunofluorescence staining (Additional file 1: Fig. S3), and further evaluated by flow cytometry analysis in the adherent and suspension cells at different time points, thus demonstrating the acquired hematopoietic phenotype (Fig. 4D). Without doubt, the above results provided the strong evidence of the hematopoietic development, thus supporting our differentiation model.

### hESC-derived CD45^+^ cells showed hematopoietic potential in vitro

**Fig. 5:**
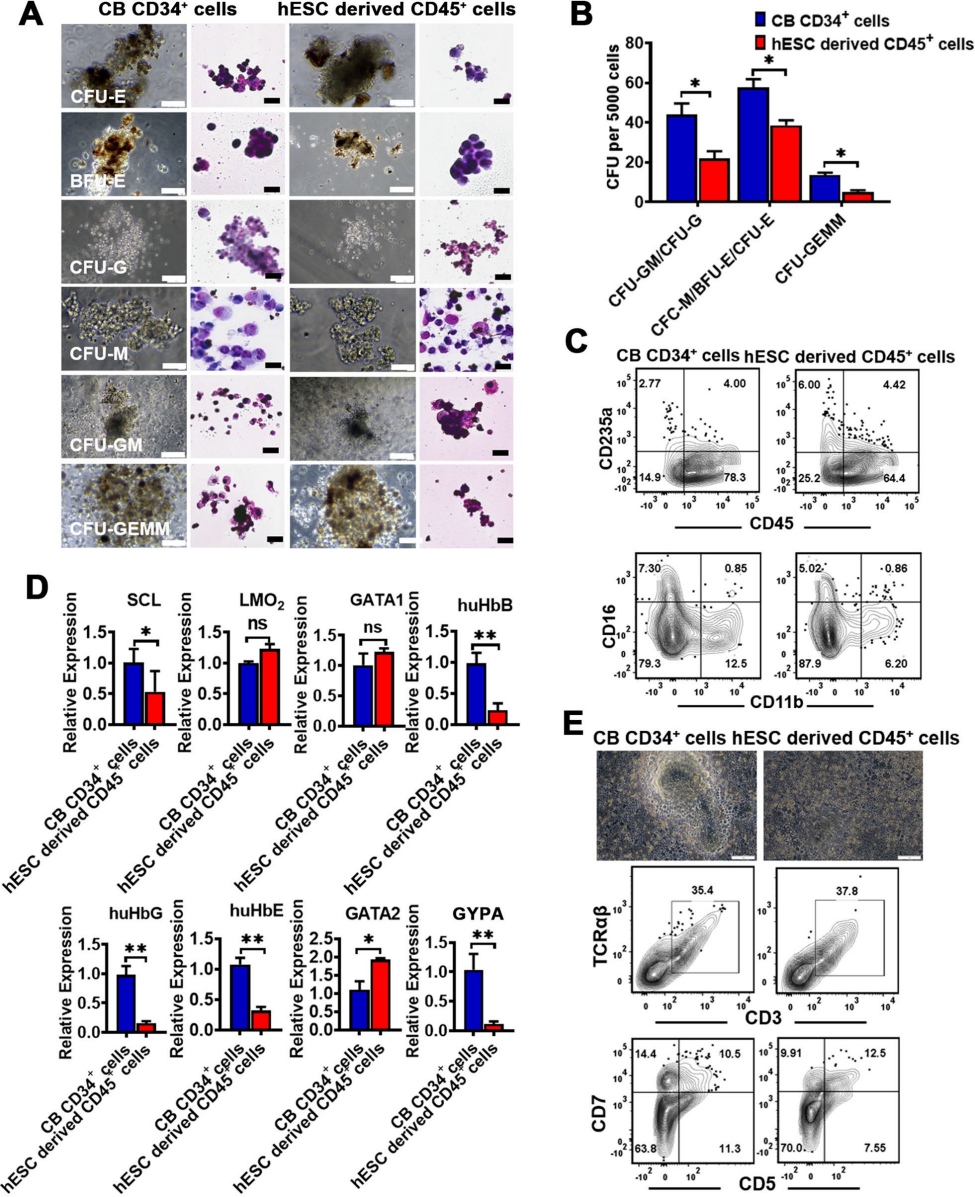
Hematopoietic potential of the hESC-derived CD45^+^ cells in vitro. **A** CB CD34^+^ cells and CD45^+^ cells derived from hESCs at day 15 after differentiation from hESCs were collected for MethoCult colony assays. **B** The colony-forming potential of CD45^+^ cells derived from hESCs was compared with those from CB CD34^+^ cell populations, and the number of CFU-GEMM, CFU-E and CFU-G/M was scored 2 weeks after the colony-forming. **C** All colonies were isolated and surface phenotypes of the cells from these colonies was assessed by flow cytometry. **D** Colonies were isolated for analyzing the expression of megakaryocytic gene (LMO2, SCL and GATA2), erythroid genes (huHbB, huHbG and huHbE), granulocyte (GYPA) and HPCs genes (GATA1) by RT-qPCR (*n* = 3, mean ± SEM). **E** T cell potential of HPCs generated from hESCs at day 15 after differentiation from hESCs following 4 weeks of inductive culture for production of T cells. Data represent the mean ± SEM. *p < 0.05, **p < 0.01, and ns (no significance)

hESC-derived CD45^+^ cells demonstrated clonogenic potential in a methylcellulose assay and gave rise to different colony types as CB CD34^+^ cells did. Among these colonies, they contained almost all kinds of hematopoietic colonies, including erythroid CFU (CFU-E), erythroid burst-forming unit (BFU-E), granulocyte CFU (CFU-G), macrophage CFU (CFU-M), granulocyte/macrophage CFU (CFU-GM) and granulocyte/erythroid/macrophage/megakaryocyte CFU (CFU-GEME) (Fig. 5A, B). Using flow cytometry, we identified that hematopoietic colonies were composed of CD45^+^ cells that contained CD235a^+^ erythroids, CD16^+^ granulocytes and CD11b^+^ myeloid cells, characteristic of hematopoietic cells (Fig. 5C). RT-qPCR analysis also confirmed this result (Fig. 5D); briefly, concordant with the colonies produced by CB CD34^+^ cells, the colonies produced by hESC-derived CD45^+^ cells expressed a considerable amount of megakaryotic genes (LMO2, SCL and GATA2), erythroid gene (huHbB, huHbG and huHbE), granulocyte (GYPA) and HPCs genes (GATA1). More importantly, T lineage potential is a vitally important hallmark of definitive hematopoiesis. To evaluate the production of lymphoid hematopoietic potential, hESC-derived CD45^+^ cells and CB CD34^+^ cells were, respectively, collected and co-cultured with OP9 stroma cell in a gas–liquid culture system. We found that hESC-derived CD45^+^ cells with cocultures of OP9 cells gave rise to about 37.8% CD45^+^CD3^+^TCRαβ^+^ T cells, very similar to the efficiency of CB CD34^+^ cells did in vitro (Fig. 5E). Moreover, we could also detect a significant T-cell progenitor population (CD45^+^CD5^+^ cells and CD45^+^CD7^+^ cells) in both two groups (Fig. 5E), which demonstrated the generation of human lymphoid hematopoiesis in vitro. Together, these results strongly demonstrated that hESC-derived CD45^+^ cells labeled definitive multipotent HPCs from the differentiation of hESCs (hESC-derived HPCs).

### hESC-derived CD45^+^ cells exhibited an engraftment potential for hematopoietic multi-lineages in vivo

**Fig. 6:**
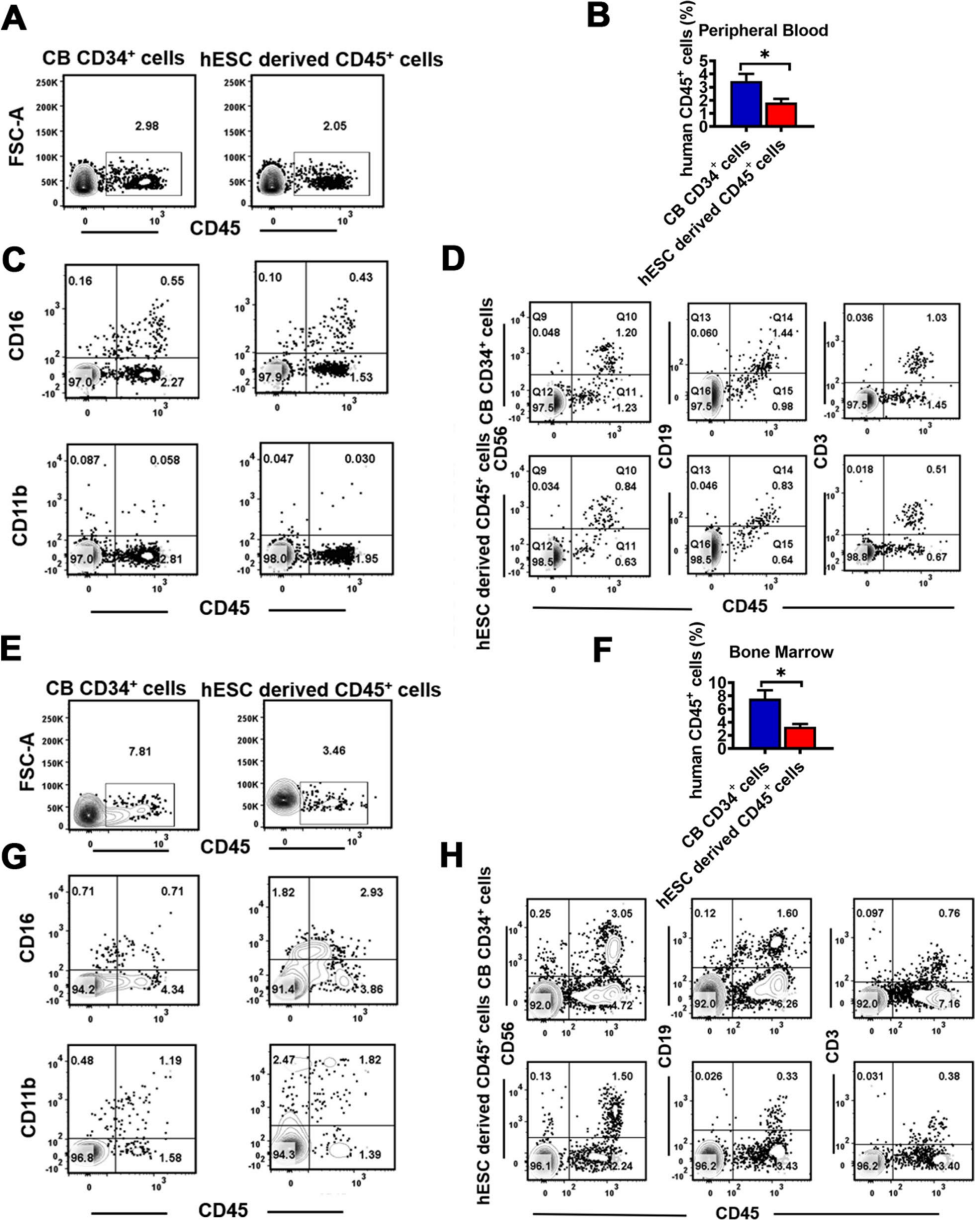
In vivo lineage potential of hESC-derived CD45^+^ cells. **A** and **B** Human CD45^+^ cells were measured by flow cytometry in peripheral blood of the mice transplanted with CB CD34^+^ cells and hESC-derived CD45^+^ cells at one month post-transplantation. **C** and **D** Myeloid lineage **C** and lymphoid lineage **D** contribution by CB CD34^+^ cells and hESC-derived CD45^+^ cells in peripheral blood of engrafted mice at one month post-transplantation. Flow analysis for myeloid cells (CD11b), granulocyte cells (CD16), B cells (CD19), NK (CD56) and T cells (CD3) within the human CD45^+^ population. **E** and **F** Human CD45^+^ cells were measured by flow cytometry in bone marrow in the mice transplanted with CB CD34^+^ cells and hESC-derived CD45^+^ cells at one month post-transplantation. **G** and **H** Myeloid lineage **E** and lymphoid lineage **H** contribution by CB CD34^+^ cells and hESC-derived CD45^+^ cells in bone marrow of engrafted mice at one month post-transplantation. Flow analysis for myeloid cells (CD11b), granulocyte cells (CD16), B cells (CD19), NK (CD56) and T cells (CD3) within the human CD45^+^ population. Data represent the mean ± SEM. *p < 0.05

We next aimed to identify whether hESC-derived CD45^+^ cells promoted hematopoietic reconstitution, and hESC-derived CD45^+^ cells and CB CD34^+^ cells were injected via tail vein into NCG-X mice with T/B/NK cell immunodeficiency plus the knockout of c-kit allele. Then, in vivo reconstitution efficacy was monitored for the percentage of human CD45^+^ cells in PB and BM of the recipient xenograft animals by staining with a human-specific CD45 antibody. At one month post-transplantation, analysis of PB chimerism showed a similar level of human white blood cells derived from CB CD34^+^ cells (2.98%) in treated mice to those from hESC-derived CD45^+^ cells (2.05%) (Fig. 6A, B). The engrafted human CD45^+^ cells in mice treated with from hESC-derived CD45^+^ cells contained a number of proportion of CD16^+^ granulocytes (0.43%) and CD11b^+^ myelocyte (0.03%) as well as lymphoid lineages such as CD19^+^ B cells (0.83%), CD56^+^ NK cells (0.84%) and CD3^+^ T cells (0.51%), very similar to those in PB of the mice transplanted with the CB CD34^+^ cells (Fig. 6C, D). Moreover, we also detected a significant higher number of human CD45^+^ cells from the BM than that from PB in the mice transplanted with hESC-derived CD45^+^ cells one month after transplantation (Fig. 6E, F). The engrafted human CD45^+^ cells varied from near 3.46% of total cells and contained a certain proportion of myeloid and lymphoid lineage cells, indicating that hESC-CD45^+^ cells exhibited the capacity of homing to BM as CB CD34^+^ cells did after the transplantation in vivo (Fig. 6G, H). The engraftment capability of hESC-derived CD45^+^ cells into immunodeficient mice constitutes an important step in the right direction for the field, to some extent, demonstrating a completely hematopoietic multilineage program appeared to be established. Future investigation should be taken into consideration to identify what inductive signals may endow this capability.

### Hypoxia increased the efficiency of the differentiation toward HPCs by activating the arterialization-related signaling

**Fig. 7:**
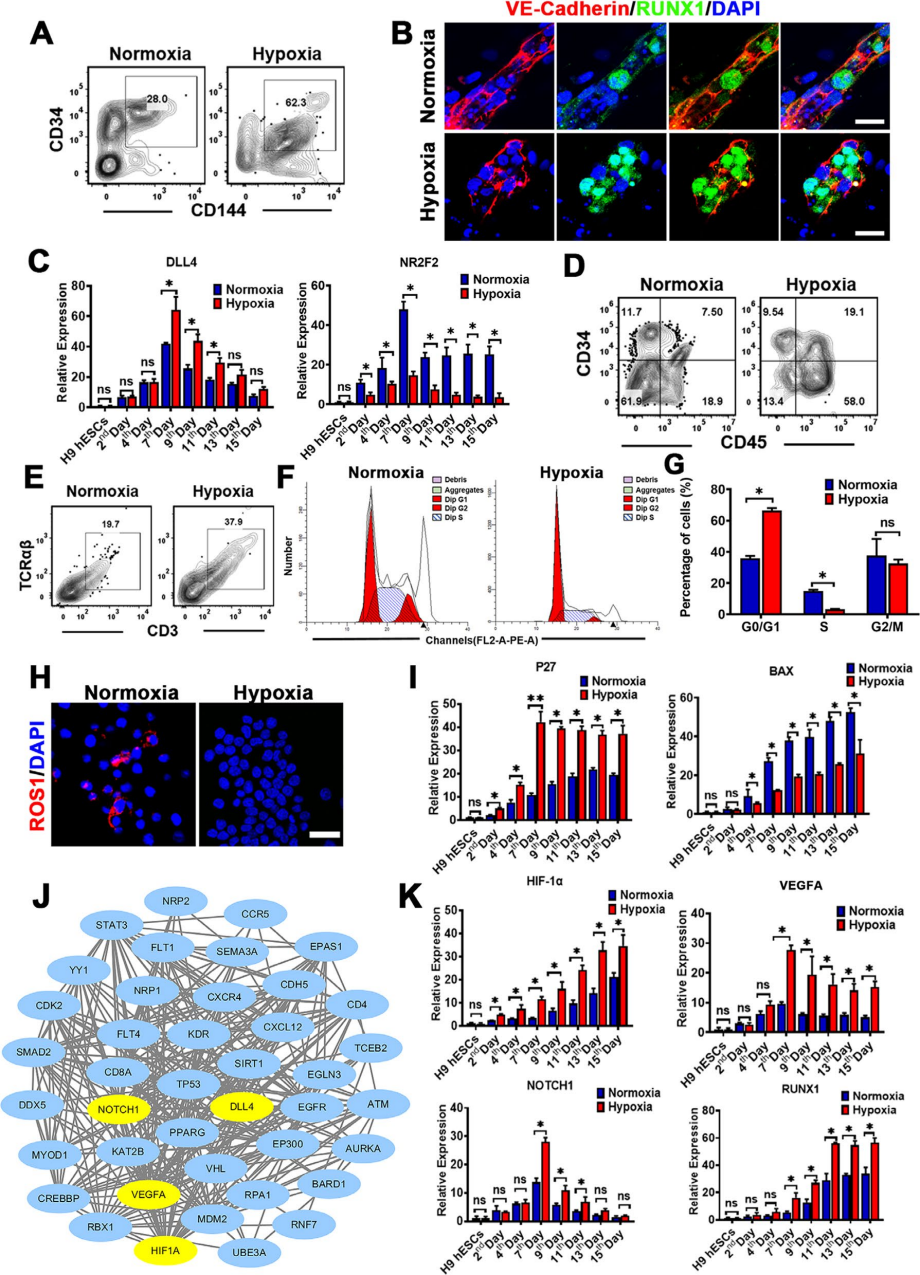
Hypoxia enhanced the hematopoietic differentiation by promoting the EHT progression. **A** Flow cytometry analysis for HEPs (hESC-derived CD144^+^CD34^+^ cells) at day 11 after the hematopoietic differentiation under the hypoxia or normoxia conditions. **B** CLSM images of the immunostaining for VE-Cadherin and RUNX1 at day 15 cells during the hematopoietic specification under the normoxia or hypoxia conditions. The colors of green, red and blue represent the staining for RUNX1, VE-Cadherin and nuclei. (Original magnification, 40×, scale bar 50 μm). **C** Relative endothelial gene expression of differentiated cells at different time points under the normoxia or hypoxia conditions. **D** Flow cytometry analysis for HPCs (hESC-derived CD34^+^CD45^+^ cells) during the hematopoiesis under the hypoxia or normoxia conditions. **E** T lineage potential of HPCs at day 15 after the hematopoietic differentiation following 4 weeks of culture for induction of T cells. **F** and **G** Cell cycle distribution was determined by flow cytometry after the hematopoietic differentiation under the normoxia or hypoxia conditions. The percentage of S phase was significantly increased in hESC-derived CD34^+^CD45^+^ cells under the normoxia conditions. **H** CLSM images of the immunostaining for ROS1 in hESC-derived CD34^+^CD45^+^ cells under the normoxia or hypoxia conditions. The colors of red and blue represent the staining for ROS1 and nuclei. (Original magnification, 40×, scale bar 50 μm). **I** Gene expression analysis of cell cycle gene (P27) and apoptosis gene (BAX) by RT-qPCR at different time points during the hematopoietic differentiation under the normoxia or hypoxia conditions (*n* = 3, mean ± SEM). **J** Gene set enrichment analysis for the identified genes involved in HIF-1a induced hypoxia during the hematopoietic specification. **K** RT-qPCR analysis for the expressions of HIF-1α, VEGFA, NOTCH1 and RUNX1. Data represent the mean ± SEM. *p < 0.05, **p < 0.01, and ns (no significance)

Previous studies have shown that the hypoxia could drive the hematopoietic differentiation [15]. The occurrence of the EHT in vitro closely mimics how hematopoietic cells develop in vivo, although this process is accompanied by gradual downregulation of aerobic metabolism; however, it still remains in highly active aerobic metabolism when compared to those in vivo [15]. To experimentally demonstrate the acquisition of the hematopoiesis under the hypoxia conditions, hESCs were therefore induced to differentiate into hematopoietic lineage under the hypoxia (5%) or the normoxia (21%) conditions. Then, we analyzed HEPs differentiation by flow cytometry for surface markers CD144 and CD34 and found that the differentiation efficiency for HEPs was significantly increased under the hypoxia condition (Fig. 7A). This observation was further confirmed by co-immunostaining of both VE-Cadherin and RUNX1 (Fig. 7B). Moreover, during the EHT phase, it was found that arterialization-related signaling genes DLL4 were significantly activated, and venous endothelium-related gene NR2F2 was downregulated, suggesting that the hypoxia enhanced hematopoietic differentiation by promoting arterialization (Fig. 7C). We further confirmed this enhanced effect on the differentiation outcome by additionally analyzing surface markers CD144 and CD34, the double-positive population for CD144 and CD34 contained HEPs (Fig. 7D). More importantly, T lineage induction showed that the hypoxia significantly promoted the generation of HPCs with a higher population of CD45^+^CD3^+^TCRαβ^+^ T cells, indicating a strong tendency to T lineage differentiation (Fig. 7E). Even brief exposure to high-oxygen environment could have significantly negative effects on HSCs by reducing the number of HSCs and accumulating excessive reactive oxygen species (ROS), and ultimately decreasing its function; furthermore, this condition destroys the resting state of HSCs and makes the loss of the ability of self-renewal [19]. Then, we assessed the cell cycle distribution of hESC-derived CD45^+^ cells differentiated from the hypoxia or normoxia conditions and found that significantly decreased population in S phase was observed in the hypoxia group in comparison with those in the normoxia group (Fig. 7F, G), indicating that the cell cycling was delayed and the cells maintained the resting state. As expected, immunofluorescence staining further confirmed that this effect of ROS1 accumulation could be attenuated under the hypoxia condition, thus protecting the hematopoiesis from the oxidative stress response (Fig. 7H). The above results were also verified by RT-qPCR analysis for the expression of some important genes P27 (an antagonist of cell cycle) and BAX (apoptosis-promoting gene) (Fig. 7I). HIF-1α, significantly upregulated in hESC-derived CD45^+^ cells produced under the hypoxia conditions (Fig. 7K), is identified as a transcription factor that plays an important regulatory role in angiogenesis during the development of hematopoietic lineage. To further elucidate HIF-1α-regulated pathway, we analyzed the PPI network of HIF-1α in STRING database and screened the potential proteins that strongly interacted with HIF-1α. Intriguingly, we observed that VEGFA, DLL4 and NOTCH1, which were all differentially expressed genes involved in the HIF-1α-regulated pathway, might also interact with HIF-1α (Fig. 7J). The RT-qPCR analysis results showed that the expression of RUNX1 and the above three genes were positively regulated by HIF-1α and had a positive regulation for artery activation and the production of lymphoid HPCs (Fig. 7C,K). These results indicated that VEGFA, DLL4 and NOTCH1 might serve as critical molecules involved in HIF-1α-regulated pathway for the development of the hematopoiesis under the hypoxia conditions.

## Discussion

HSC transplantation has been used widely in the treatment of hematologic and genetic disorders [1–3]. hPSCs-derived HSCs could be an ideal source for resolving current limitations related to HSCT [4]. Oxygen concentration in the normoxia and hypoxia in particular plays an important regulatory role in early embryonic development [26], and although well studied in adult hematopoiesis, the role of the hypoxia remains largely unexplored during human embryonic hematopoiesis. To eventually acquire functional HSCs, it is critical to model the regulatory mechanisms during early embryonic development. Based on this expectation, we utilized a four-stage in vitro hematopoietic differentiation from hESCs toward hematopoiesis based on hypoxia condition, which recapitulates key stages of embryonic hematopoiesis. The purpose of studying the regulatory effect of the hypoxia on hematopoietic differentiation, was to find new clues for the embryonic hematopoiesis in vitro. In order to trace the embryonic hematopoiesis in vitro, the hematopoietic-like cells expressing the mCherry driven by hematopoietic specific HOXA9 promoter were visualized to show gradually climbing out from an organized and arborizing endothelial-like network by the use of engineered hESC line with universal promoter EF1α and HOXA9 promoter driving the EGFP (green) and mCherry (red) reporters, respectively. The results of both the immunofluorescence staining and flow cytometry analysis demonstrated the generation of hematopoietic lineages from hESCs in vitro, in particular, the production of lymphoid hematopoietic potential which is very similar to CB CD34^+^ cells in vitro. One month post-transplantation, 2% human CD45^+^cells were detected in the peripheral blood of mice transplanted with hESC-derived CD45^+^cells, showing significantly reconstitution for all hematopoietic lineages in vivo*,* and our results were obviously superior to the reconstruction efficiency as previously reported [8, 27]. Targeted attention of T lineage potential was needed, as could be seen from the data above, NK cells and B cells in CD45^+^ cells were increased in BM after transplantation, T lineage reconstitution had also been detected in vivo. These results demonstrated that a complete program for hematopoietic multilineage has been established, and further investigation is necessitated to determine is what inductive signals may endow this capability.

Previous studies on the regulation of the hypoxia on hematopoiesis mainly focused on the primitive hematopoiesis occurring in the yolk sac [28]. A large number of studies have shown that HEPs are the earliest group of cells that show the potential of hematopoietic differentiation in the process of hematopoietic differentiation and development [29, 30]. VE-Cadherin and CD31 are expressed in the aorta-gonad-mesonephros region (AGM) of the hematopoietic organ that first produces HSCs in the development of danio rerio and mice by directly tracing the development process. HEPs co-expressed both CD34 and TIE2 can directly produce definitive HSCs [29, 30]. The differentiation of ES cells into hematopoietic stem/progenitor cells in mice and humans also demonstrated that definitive HSCs were derived from HEPs [29]. In this study, we found that the hypoxia could promote the differentiation of hESCs into HEPs, and these results were corroborated by observations in an important role of the hypoxia in the generation of CD144^+^CD34^+^CD45^−^ HEPs during the occurrence of the EHT, positively regulated human embryonic hematopoiesis. Hypoxia stimulation was also proved to improve human HPCs efficiency by generating CD45^+^ hematopoietic cells, which is consistent with other reports [31]. These studies have described an in vitro requirement for hypoxia signaling to properly instruct normal hematopoiesis and functional maintenance [32–34] and thus further increase the efficiency of HSC transplantation [19]. This requirement for hypoxia signaling is stage-specific. In our study, we found that the activation of HIF-1α expression positively impacted the hematopoiesis only during certain stages of the differentiation from hESCs. To mimic early embryonic hematopoiesis, specific cytokines such as BMP4 and bFGF were used to induce the differentiation of hESCs into mesoderm, and the gene expression of the mesoderm was maximized at day 4 after the differentiation. Meanwhile, HIF-1a, a downstream gene of the hypoxia, was highly expressed in the hypoxia group, demonstrating that the hypoxia pathway was activated. Mesoderm was then further induced for directional specialization toward HEPs in the presence of cytokines such as VEGF and bFGF. In the hypoxia conditions, more CD34^+^CD144^+^ HEPs were induced from hESCs than those in normoxia conditions, and the early HEPs-related gene, DLL4, was upregulated. Furthermore, cellular immunofluorescence staining also revealed that more RUNX1^+^VE-Cadherin^+^HEPs were produced along with the increased expression of the hypoxia gene HIF-1a. Therefore, our study indicates that the hypoxia regulated the hematopoiesis mainly by promoting the production of HEPs, which not only express the characteristics of endothelial cells, but also specifically express RUNX1 transcription factor (TF), an important TF to regulate the development of the hematopoiesis. These HEPs would further go on to produce definitive HSCs. Therefore, these results provided an important theoretical basis for promoting the differentiation of hESCs toward definitive hematopoiesis in the hypoxia condition.

It has been shown that hypoxia can regulate the cellular and metabolic characteristics of HPCs, including the inhibition of proliferation and quiescence, and promote the development of early lymphoid progenitor cells by upregulating the expression of hypoxia-inducible factor HIF-1/2α [35]. It has been reported that the hypoxia can activate the upregulation of arterialization-related signaling genes, which will further affect the production of HEPs during the hematopoietic differentiation, which in turn promotes the hematopoiesis [15]. Therefore, it is worth mentioning that the reason why human ESCs or iPSCs cannot produce definitive HSC may be due to the long-term neglect of the regulatory effect on arterial characteristics [36]. Therefore, activating the expression of arterial specific genes in the differentiation process is the key to produce human definitive HSC capable of the reconstitution of hematopoietic multi-lineages [37, 38]. What it more interests us is whether hypoxia can promote the generation of T lineage by activating arterialization-related signals by using our differentiation system. The results demonstrated that hypoxia indeed regulated HIF-1α signaling by upregulating arterialization-related signaling genes (such as VEGFA, DLL4 and NOTCH1), driving the process of EHT, thereby promoting the generation of T-lymphoid lineage. Under physiological conditions, HSCs are located in the low-oxygen environment with an average oxygen concentration of 3% in the human body; however, the collection and manipulation of HSCs are usually completed in the environment with a normal oxygen concentration of 21% [19]. Higher oxygen concentrations far in excess of physiological conditions has a significantly negative impact on HSCs, resulting in a decrease in the number of HSCs, ultimately reducing the function and efficacy of transplantation. As reported in the literature, HSCs/HPCs reside in the hypoxia niche and limit the production of ROS for self-protection [39, 40]. To explore whether the hypoxia could limit ROS production, we examined cellular responses as shown in Fig. 7H. Low levels of ROS and apoptosis in the hypoxia group favored enhancing HSC self-renewal. Therefore, our results demonstrated that the hypoxia not only promoted the occurrence of the EHT, but also protected HSCs from ROS damage and thus maintained a good self-renewal capacity. Further research on the role of the hypoxia in the hematopoiesis and blood diseases is of great significance to our understanding of the mechanism of hematopoiesis regulation, as well as the expansion and reconstruction of HSCs in vitro and in vivo, and can provide new insights for finding therapeutic targets for treating blood diseases.

## Conclusion

In the present study, we demonstrated that hypoxia drove the production of HEPs during the hematopoietic differentiation, which will further promote hematopoiesis. Hypoxia plays an important role in T lineage hematopoiesis by promoting the expression of arterial endothelial gene DLL4 and upregulation of NOTCH1 through the activation of the HIF-1α signaling pathway. These results of this study may provide a significant approach for in vitro and in vivo production of fully functional hematopoietic stem/progenitor cells from hESCs.

## Supplementary Information

**Additional file 1: Fig. S1.** Confocal microscopy visualization of emerging mesendoderm determined by immunofluorescence staining for mesendodermal markers SOX17 and Brachyury at day 2, 4 and 6 after the differentiation from hESCs under the hypoxia conditions. (Original magnification, 20 × , scale bar 40 μm). **Fig. S2.** Construction and validation of the lentivirus containing EGFP and mCherry driven by the universal promoter EF-1α and HSC-specific promoter HOXA9 (EF1α-EGFP-HOXA9-mCherry), respectively. (A) Schematic diagram of the lentiviral vector designated as EF1α-EGFP-HOXA9-mCherry. (B) The transduced efficiency of EF1α-EGFP-HOXA9-mCherry construct was evaluated by flow cytometry analysis for EGFP-positive population. (C) Flow analysis showed that the pluripotency of engineered hESCs was not affected by the transduction, as determined by the flow cytometry analysis for OCT4 and SSEA4. **Fig. S3.** Confocal microscopy visualization of emerging hematopoietic cells determined by immunofluorescence staining for hematopoietic markers CD34 and CD45 at day 11 after the differentiation from hESCs under the hypoxia conditions. (Original magnification, 20 × , scale bar 40 μm). **Table S1.** Antibodies used in this study. **Table S2.** Primers for mRNA qPCR.

## Data Availability

The data that support the findings of this study are available from the corresponding author upon reasonable request.

## Abbreviations

hPSCs: Human pluripotent stem cells
hESC: Human embryonic stem cell
HSCs: Hematopoietic stem cells
HSCT: HSC transplantations
HEPs: Hematopoietic endothelial progenitors
HPCs: Hematopoietic progenitor cells
EHT: Endothelial-to-hematopoietic transition
