# Supplementary material for: Hypoxia drives hematopoiesis with the enhancement of T lineage through eliciting arterial specification of hematopoietic endothelial progenitors from hESC
Source: Stem Cell Res Ther. 2022 Jun 28;13:282. doi: 10.1186/s13287-022-02967-0 (PMC9241298; doi:10.1186/s13287-022-02967-0)
Supplement: Supplementary file 1 — Additional file 1: Fig. S1. Confocal microscopy visualization of emerging mesendoderm determined by immunofluorescence staining for mesendodermal markers SOX17 and Brachyury at day 2, 4 and 6 after the differentiation from hESCs under the hypoxia conditions. (Original magnification, 20 × , scale bar 40 μm). Fig. S2. Construction and validation of the lentivirus containing EGFP and mCherry driven by the universal promoter EF-1α and HSC-specific promoter HOXA9 (EF1α-EGFP-HOXA9-mCherry), respectively. (A) Schematic diagram of the lentiviral vector designated as EF1α-EGFP-HOXA9-mCherry. (B) The transduced efficiency of EF1α-EGFP-HOXA9-mCherry construct was evaluated by flow cytometry analysis for EGFP-positive population. (C) Flow analysis showed that the pluripotency of engineered hESCs was not affected by the transduction, as determined by the flow cytometry analysis for OCT4 and SSEA4. Fig. S3. Confocal microscopy visualization of emerging hematopoietic cells determined by immunofluorescence staining for hematopoietic markers CD34 and CD45 at day 11 after the differentiation from hESCs under the hypoxia conditions. (Original magnification, 20 × , scale bar 40 μm). Table S1. Antibodies used in this study. Table S2. Primers for mRNA qPCR. [file 13287_2022_2967_MOESM1_ESM.doc]

***Supplementary Materials for***

**Hypoxia drives hematopoiesis with the enhancement of T lineage through eliciting arterial specification of hematopoietic endothelial progenitors from hESC**

Ning Wang^1,2†^, Chuxin Chen^3†^, Yang Cheng^4†^, Yingjie Fu^2^, Zhiyong Zhong^1,2^, Yu Yang^4^, Ling Lv^5*^, Honglin Chen^2,6,7,8*^, Jian Huang^9,10*^, Yuyou Duan^2,6,7,8*^

1. School of Biomedical Sciences and Engineering, Guangzhou International Campus, South China University of Technology, Guangzhou 510006, China
2. Laboratory of Stem Cells and Translational Medicine, Institutes for Life Sciences and School of Medicine, South China University of Technology, Guangzhou 510006, China
3. School of Biology and Biological Engineering, South China University of Technology, Guangzhou 510006, China
4. Department of Gynaecology and Obstetrics, Guangzhou First People's Hospital, Guangzhou 510180, China
5. Hepatobiliary Center, The First Affiliated Hospital of Nanjing Medical University, 300 Guang Zhou Road, Nanjing 210029, China
6. National Engineering Research Center for Tissue Restoration and Reconstruction, South China University of Technology, Guangzhou 510006, China
7. Guangdong Provincial Key Laboratory of Biomedical Engineering, South China University of Technology, Guangzhou 510006, China
8. Key Laboratory of Biomedical Materials and Engineering of the Ministry of Education, South China University of Technology, Guangzhou 510006, China
9. Coriell Institute for Medical Research, Camden, NJ, United [States](mailto:States;jhuang@coriell.org)
10. Cooper Medical School of Rowan University, Camden, NJ, United States

^†^Ning Wang, Chuxin Chen and Yang Cheng contributed equally to this work.

***Correspondence to:**

Yuyou Duan, Ph.D.

Laboratory of Stem Cells and Translational Medicine

Institutes for Life Sciences

School of Medicine

South China University of Technology

No.382 Waihuan East Road, Suite 406

Higher Education Mega Center

Guangzhou 510006, P.R.China

Telephone: +862039390970

E-mail: [yuyouduan@scut.edu.cn](mailto:yuyouduan@scut.edu.cn)

Jian Huang, Ph.D.

Coriell Institute for Medical Research

Camden, NJ, United [States](mailto:States;jhuang@coriell.org)

1. mail: [jhuang@coriell.org](mailto:jhuang@coriell.org)

Honglin Chen, Ph.D.

Laboratory of Stem Cells and Translational Medicine

Institutes for Life Sciences

School of Medicine

South China University of Technology

382 Waihuan East Road, Suite 406

Higher Education Mega Center

Guangzhou 510006, P.R.China

Telephone: +862039390970

E-mail: [chlolinba@outlook.com](mailto:chlolinba@outlook.com)

Ling Lv, Ph.D.

Hepatobiliary Center

The First Affiliated Hospital of Nanjing Medical University

Research Unit of Liver Transplantation and Transplant Immunology

Chinese Academy of Medical Sciences

Nanjing 210029, P.R.China

E-mail: [lvling@njmu.edu.cn](mailto:lvling@njmu.edu.cn)

**Supplementary Figures**


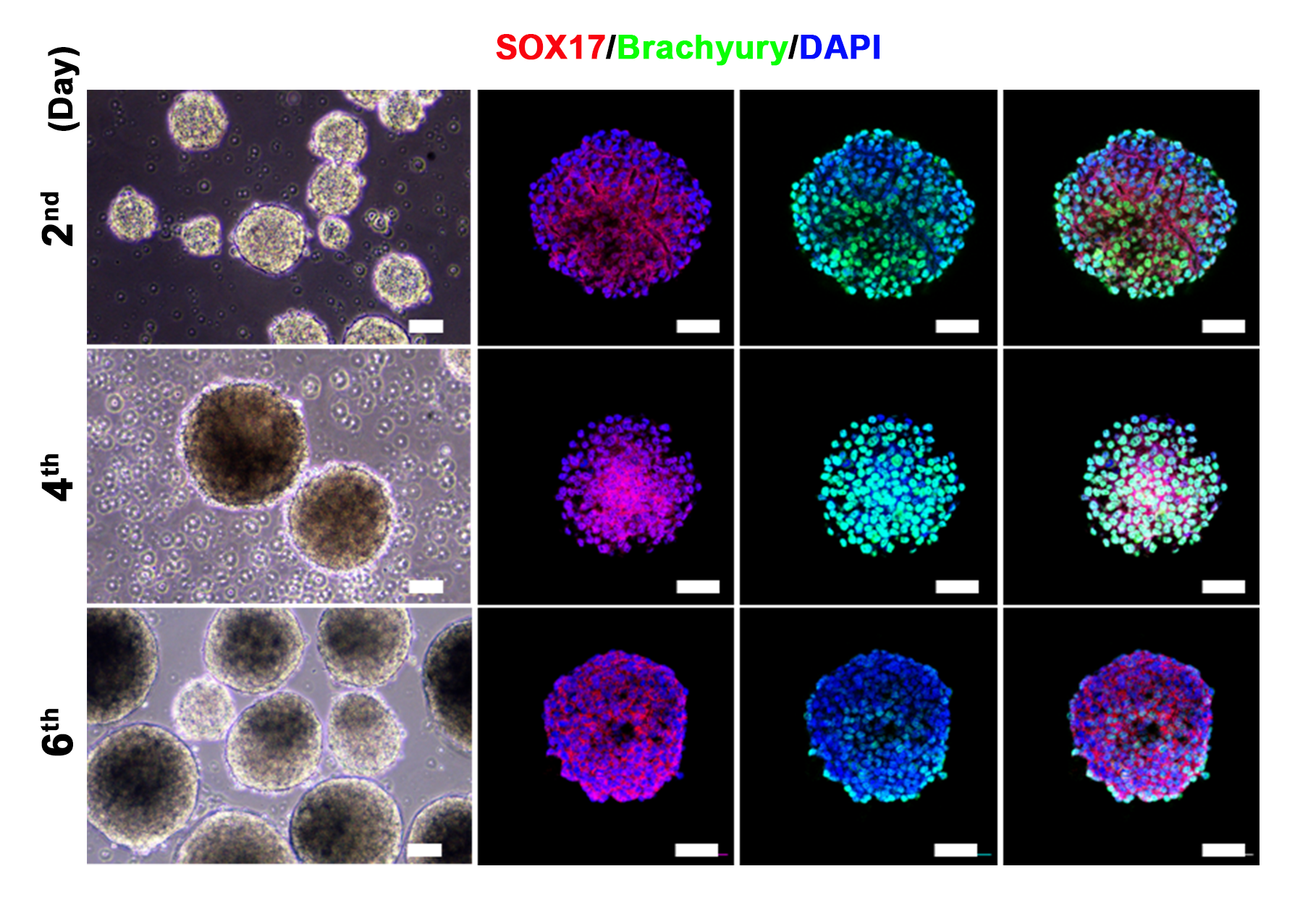


**Fig. S1** Confocal microscopy visualization of emerging mesendoderm determined by immunofluorescence staining for mesendodermal markers SOX17 and Brachyury at day 2, 4 and 6 after the differentiation from hESCs under the hypoxia conditions. (Original magnification, 20×, scale bar 40 μm).


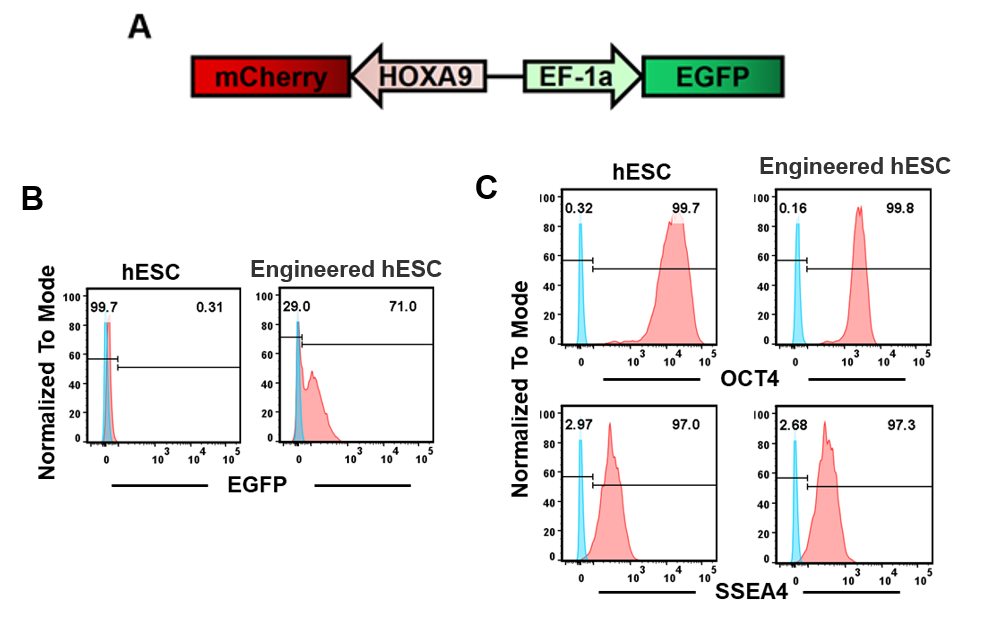


**Fig. S2** Construction and validation of the lentivirus containing EGFP and mCherry driven by the universal promoter EF-1α and HSC-specific promoter HOXA9 (EF1α-EGFP-HOXA9-mCherry) respectively. (A) Schematic diagram of the lentiviral vector designated as EF1α-EGFP-HOXA9-mCherry. (B) The transduced efficiency of EF1α-EGFP-HOXA9-mCherry construct was evaluated by flow cytometry analysis for EGFP positive population. (C) Flow analysis showed that the pluripotency of engineered hESCs was not affected by the transduction, as determined by the flow cytometry analysis for OCT4 and SSEA4.


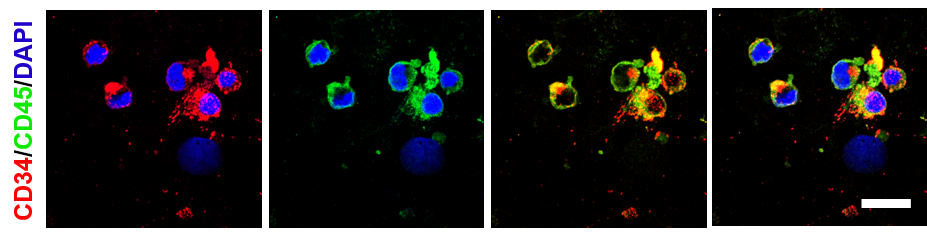


**Fig. S3** Confocal microscopy visualization of emerging hematopoietic cells determined by immunofluorescence staining for hematopoietic markers CD34 and CD45 at day 11 after the differentiation from hESCs under the hypoxia conditions. (Original magnification, 20×, scale bar 40 μm).

Supplementary Tables

**Table S1.** Antibodies used in this study

| **Name** | **Species reactivity** | **Providers** | **Cat. No.** | |
| --- | --- | --- | --- | --- |
| FITC anti-human CD45 Antibody | human | BioLegend | | 304006 |
| APC/Cyanine7 anti-human CD45 Antibody | human | BioLegend | | 304014 |
| PE/Dazzle™ 594 anti-human CD34 | human | BioLegend | | 343534 |
| PE anti-human CD34 | human | BioLegend | | 343606 |
| FITC anti-human CD43 Antibody  Alexa Fluor 700 anti-human CD31  PE Mouse Anti-Human CD309 (VEGFR-2)  PE Anti-Human SSEA-4 Antibody  BV421 -CD235a  BV421-TCRαβ  PE/Cy7 anti-human CD11b  PerCP/Cyanine5.5 anti-human CD16  PE/Cy5 anti-human CD56 (NCAM)  Brilliant Violet 785™ anti-human CD19  PE anti-human CD326 (Ep-CAM)  Brilliant Violet 510™ anti-human CD3  FITC anti-human CD5  PE anti-human CD7  APC anti-human CD144  VEGFR2/KDR/Flk-1 Antibody  CD31 (PECAM-1) (89C2) Mouse mAb  CD31 (PECAM-1) Antibody  CD34 Antibody | human  human  human  human  human  human  human  human  human  human  human  human  human  human  human  human  human  human  human | BioLegend  BioLegend  BD  STEM CELL  BD  BioLegend  BioLegend  BioLegend  BioLegend  BioLegend  BioLegend  BioLegend  BioLegend  BioLegend  BioLegend  RD  CST  ReliaTech GmbH | | 315204  303134  560494  60062PE  562938  306722  301321  302028  362516  302240  324206  317332  364022  343106  348508  MAB3571  3528S  102-PA07S  ab54208 |
| CD45 (D9M8I) XP Rabbit mAb  Human RUNX1/CBFA2 MAb  VE-Cadherin (D87F2) XP® Rabbit mAb  Anti-mouse IgG (H+L) F(ab')2 Fragment (Alexa Fluor 594 Conjugate)  Anti-rabbit IgG (H+L) F(ab')2 Fragment (Alexa Fluor 594 Conjugate)  Anti-mouse IgG (H+L)F(ab')2 Fragment (Alexa Fluor 488 Conjugate)  Anti-rabbit IgG (H+L)F(ab')2 Fragment (Alexa Fluor 488 Conjugate) | human  human  human  /  /  /  / | Abcam  RD  CST  CST  CST  CST  CST | | 13917T  MAB23991  2500S  8890S  8889S  4408S  4412S |

**Table S2. Primers for mRNA qPCR**

| **Name** | **Sequences** | **Providers** |
| --- | --- | --- |
| BMP4-qF | TAGCAAGAGTGCCGTCATTCC | Sangon Biotech |
| BMP4-qR | GCGCTCAGGATACTCAAGACC | Sangon Biotech |
| KDR-qF  KDR-qR  CXCR4-qF  CXCR4-qR  CDX4-qF  CDX4-qR  DLL4-qF  DLL4-qR  Notch1-qF  Notch1-qR  EPHB4-qF  EPHB4-qR  NR2F2-qF  NR2F2-qR  GATA1-qF  GATA1-qR  Spi1-qF  Spi1-qR  HOXA9-qF  HOXA9-qR  RUNX1-qF  RUNX1-qR  LMO2-qF  LMO2-qR  SCL-qF  SCL-qR  GATA2-qF  GATA2-qR  huHbB-qF  huHbB-qR  huHbG-qF  huHbG-qR  huHbE-qF  huHbE-qR  GYPA-qF  GYPA-qR  CD56-qF  CD56-qR  Epcam-qF  Epcam-qR  T-qF  T-qR  SOX2-qF  SOX2-qR  OCT4-qF  OCT4-qR  SOX17-qF  SOX17-qR  PTPRC-qF  PTPRC-qR  P27-qF  P27-qR  BAX-qF  BAX-qR  HIF-1α-qF  HIF-1α-qR  VEGFA-qF  VEGFA-qR  GAPDH-qF  GAPDH-qR | AACGTGTCACTTTGTGCAAGA  TTCCATGAGACGGACTCAGAA  AACCAGCGGTTACCATGGAG  CACGGAAACAGGGTTCCTTCA  CGAGAAGACTGGAGCGTGTA  CTGTAGTCGGTCGAGCAGAA  GTTATTGGATGAGCAAACCAGC  AGGCAGGACAAGTTGCCATCTG  CGCTGACGGAGTACAAGTG  GTAGGAGCCGACCTCGTTG  CGCACCTACGAAGTGTGTGA  GTCCGCATCGCTCTCATAGTA  AACCAGCCGACGAGATTCG  CCCGGATGAGGGTTTCGATG  CTTGTAGTAGAGGCCGCAGG  GCTCTACCCTGCCTCAACTG  GGATGGCTGGATGGATGAGTGATACC  TGCACGCCTGTAACATCCAGC  GCGCCTTCTCCGAAAACAAT  CCAGCGTCTGGTGTTTTGTG  CTGCTCCGTGCTGCCTAC  AGCCATCACAGTGACCAGAGT  TGCCGGAGAGACTATCTCAGG  TCATCCCATTGATCTTAGTCCAC  ATGGTGCAGCTGAGTCCTCC  TCTCATTCTTGCTGAGCTTCTTG  TACCACAAGATGAATGGGCA  TCTCCTGCATGCACTTTGAC  CTG AGG AGA AGT CTG CCG TTA  AGC ATC AGG AGT GGA CAG AT  TGG ATG ATC TCAAGG GCA C  TCA GTG GTA TCT GGA GGA CA  GCA AGA AGG TGC TGA CTT CC  ACC ATC ACGTTA CCC AGG AG  GGATGTGAGGGAATTTGTCTTTTGCA  CAGTGACAGGTCCCCTAAAATGGGTTA  TCTGGATGGGCACATGGTG  TGCTCTTCAGGGTCAGCGA  CGCAGCTCAGGAAGAATGTG  TGAAGTACACTGGCATTGACG  TGTCCCAGGTGGCTTACAGATGAA  GGTGTGCCAAAGTTGCCAATACAC  GAGCTTTGCAGGAAGTTTGC  GCAAGAAGCCTCTCCTTGAA  CGACCATCTGCCGCTTTGAG  CCCCCTGTCCCCCATTCCTA  GTGGACCGCACGGAATTTG  GAGGCCCATCTCAGGCTTG  AACAGTGGAGAAAGGACGCA  TGTGTCCAGAAAGGCAAAGC  TGCAACCGACGATTCTTCTACTCAA  CAAGCAGTGATGTATCTGATAAACAAGGA  CTCACCGCCTCACTCACC  ACCCCCTCAAGACCACTC  GAACGTCGAAAAGAAAAGTCTCG  CCTTATCAAGATGCGAACTCACA  AGGGCAGAATCATCACGAAGT  AGGGTCTCGATTGGATGGCA  GAAGATGGTGATGGGATTTC  GAAGGTGAAGGTCGGAGTC | Sangon Biotech  Sangon Biotech  Sangon Biotech  Sangon Biotech  Sangon Biotech  Sangon Biotech  Sangon Biotech  Sangon Biotech  Sangon Biotech  Sangon Biotech  Sangon Biotech  Sangon Biotech  Sangon Biotech  Sangon Biotech  Sangon Biotech  Sangon Biotech  Sangon Biotech  Sangon Biotech  Sangon Biotech  Sangon Biotech  Sangon Biotech  Sangon Biotech  Sangon Biotech  Sangon Biotech  Sangon Biotech  Sangon Biotech  Sangon Biotech  Sangon Biotech  Sangon Biotech  Sangon Biotech  Sangon Biotech  Sangon Biotech  Sangon Biotech  Sangon Biotech  Sangon Biotech  Sangon Biotech  Sangon Biotech  Sangon Biotech  Sangon Biotech  Sangon Biotech  Sangon Biotech  Sangon Biotech  Sangon Biotech  Sangon Biotech  Sangon Biotech  Sangon Biotech  Sangon Biotech  Sangon Biotech  Sangon Biotech  Sangon Biotech  Sangon Biotech  Sangon Biotech  Sangon Biotech  Sangon Biotech  Sangon Biotech  Sangon Biotech  Sangon Biotech  Sangon Biotech  Sangon Biotech  Sangon Biotech |
